# A new perspective on radiotherapy in the comprehensive treatment of cancer

**DOI:** 10.1002/pro6.70073

**Published:** 2026-05-21

**Authors:** Yingze Ma, Hongxuan Yu, Shijie Shang, Shixin Shan, Dawei Chen

**Affiliations:** ^1^ Shandong University Cancer Center Shandong University Jinan Shandong China; ^2^ Department of Radiation Oncology Shandong Provincial Key Laboratory of Precision Oncology, Shandong Cancer Hospital and Institute, Shandong First Medical University, Shandong Academy of Medical Sciences Jinan Shandong China; ^3^ Cancer Center, Union Hospital Tongji Medical College, Huazhong University of Science and Technology Wuhan China; ^4^ Shandong Provincial Center for Disease Control and Prevention Jinan China

**Keywords:** combination therapy, future directions, historical development, radiotherapy

## Abstract

Radiotherapy (RT) has become an indispensable part of modern cancer treatment owing to its efficacy, precision, and safety. With continuous advances in clinical practice, the role of RT in comprehensive cancer treatment has been repositioned. Through a combination of chemotherapy, targeted therapy, and immunotherapy, RT can achieve multiple therapeutic goals, including disease cure, symptom relief, improved quality of life, and prolonged survival. Therefore, understanding the development of RT, its mechanisms, technological innovations, combined therapeutic strategies, and prospects is essential for advancing tumor treatment and improving patient survival.

## Graphical abstract

Radiotherapy remains a key component in the comprehensive management of cancer. Its effectiveness is significantly improved through strategic combinations with chemotherapy, targeted therapy, and immunotherapy, providing a range of therapeutic outcomes from cure to palliative care. Ongoing technological advances further enhance its precision, strengthening its vital role in improving patient survival and quality of life.

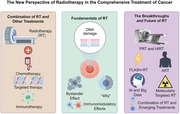

Malignant tumors are the leading cause of death worldwide. Radiotherapy (RT), which utilizes various forms of ionizing radiation such as X‐rays, γ‐rays, β‐rays, electron beams, protons, and heavy ions, is one of the three primary cancer treatments, alongside surgery and chemotherapy. In recent years, advances in computer technology, diagnostic imaging, medical physics, and biology have ushered in a new era of precision tumor treatment. While focusing on clinical needs, interdisciplinary efforts are driving the development of low‐toxicity, high‐efficiency precision RT technologies, thus consolidating the central role of RT in the treatment of tumors.

Approximately 60–70% of patients with cancer receive RT during their treatment. With the continuous advancement of RT, its application has become more widespread, offering more treatment options and improving the quality of life of patients with cancer. This article reviews and discusses the past, present, and future of RT, with a focus on its role in the comprehensive treatment of tumors.

## 1 HISTORY AND FUNDAMENTALS OF RT

### 1.1 The evolution of RT

**FIGURE 1:**
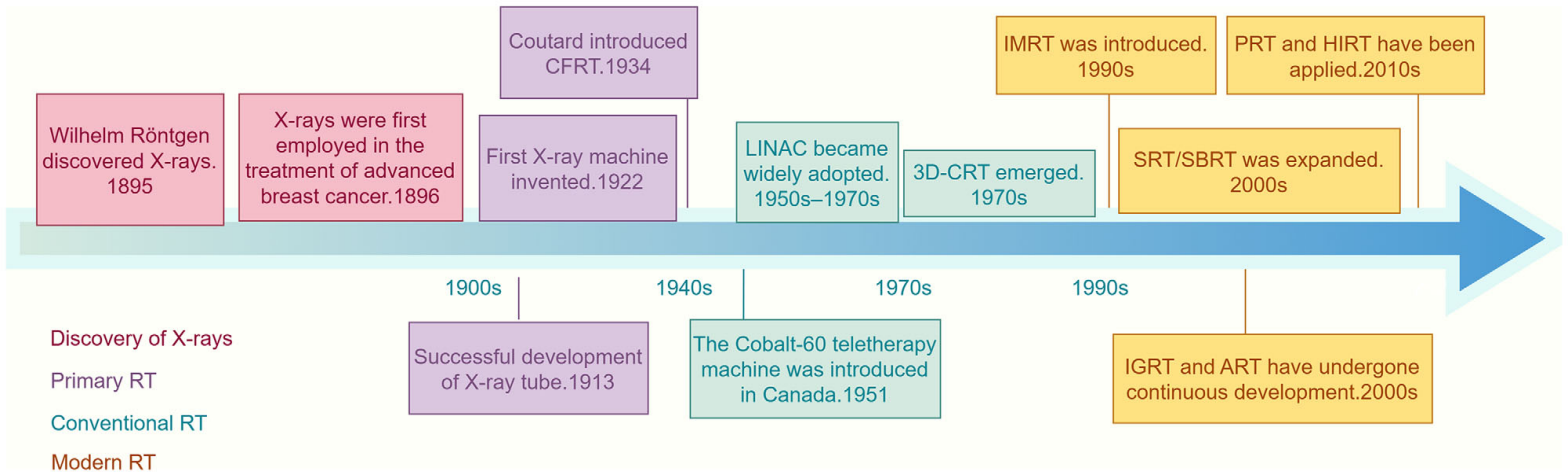
Historical timeline of important developments in RT. RT, Radiotherapy.

RT has been a cornerstone of tumor treatment for over a century. Its development occured in several stages (Figure 1).

#### 1.1.1 Discovery of X‐rays

The late 19^th^ century was a pivotal period for the development of RT. In 1895, the German physicist Wilhelm Röntgen discovered X‐rays.^1^, ^2^ In 1896, X‐rays were first used to treat advanced breast cancer.^3^ In the same year, Henri Becquerel discovered natural radioactivity.^4^ In 1898, the Polish‐born French scientists Marie and Pierre Curie isolated radionuclide radium and first introduced the concept of “radioactivity.” This concept laid the foundation for subsequent advancements in diagnostic and therapeutic radiology.^5^ In 1899, ionizing radiation was first used to treat skin cancer in Stockholm, Sweden. In 1905, Marie Curie and other scientists developed a method for encapsulating radium in platinum to create a tubular radiation source.^6^ This innovation paved the way for the treatment of skin and cervical cancers and contributed to the development of brachytherapy and intracavitary RT. However, the limitations of natural radiation sources, which include low energy, poor controllability, and shallow penetration depth, severely hampered their clinical application.

#### 1.1.2 Primary RT

Contemporary literature identifies three distinct phases in the historical evolution of RT, beginning with the initial formative phase (1900–1949). In 1913, the successful development of the X‐ray tube enabled the generation of radiation with controllable quality and quantity. This led to the creation of the X‐ray machine in 1922. The isodose chart was first used in 1923. In 1934, Coutard introduced conventional fractionation radiotherapy (CFRT), establishing it as a foundational paradigm in radiation oncology.^7^ In 1936, Moottramd et al. highlighted the critical role of oxygen in radiosensitivity, thereby establishing the foundation for radiobiological research and RT mechanism studies. Furthermore, the introduction of Roentgen (R) as a standardized radiation unit significantly advanced dosimetric precision in RT. During this pioneering era, treatment equipment primarily consisted of orthovoltage and superficial X‐ray therapy units and radium ingots. These early technologies were primarily applicable to superficial tumors and exhibited limited efficacy for deep‐seated malignancies while frequently causing collateral damage to normal tissue with suboptimal tumor control rates.

#### 1.1.3 Conventional RT

RT underwent rapid advancements during its secondary developmental phase from the 1950s to 1990s. In 1951, the Cobalt‐60 teletherapy machine was introduced in Canada^8^ to deliver high‐dose radiation (45–60 Gy) to deep tumors, while sparing superficial tissues. This machine was successfully used to treat Hodgkin lymphoma (HL) clinically, representing a major innovation in tumor treatment. Between the 1950s and 1970s, several types of accelerators were introduced. The medical linear accelerator (LINAC), which could produce different energies of X‐rays and electron beams, offered higher energy and intensity and gradually replaced conventional X‐ray machines and Cobalt‐60 machines. In the 1970s, with the advancements in computer technology, multileaf collimators (MLC) and three‐dimensional collimator treatment planning systems were developed. RT technology moved from two‐dimensional to three‐dimensional planning. Based on CT‐guided imaging, three‐dimensional conformal radiation therapy (3D‐CRT) allowed for the creation of a three‐dimensional tumor structure and the positioning of irradiation fields from different angles. This approach ensured that the high‐dose area closely matched the tumor shape, thereby minimizing exposure of the surrounding normal tissues.

#### 1.1.4 Modern RT

Since the 1990s, continuous innovations in RT equipment have driven the evolution of modern RT (Phase III) that integrates imaging technologies, computer systems, and linear accelerators.^9^ Intensity‐modulated radiation therapy (IMRT) offers the advantage of adapting the shape of the irradiation field to that of the tumor target area. In addition, it allows the beam intensity to be adjusted based on the shape of the tumor and critical surrounding organs, resulting in optimal dose distribution.^10^ Volumetric modulated arc therapy (VMAT), an advanced form of IMRT, irradiates tumors using rotational intensity modulation within a defined angular range. During the rotational irradiation process, the dose rate, rotation speed of the gantry, and position of the MLC blades were dynamically adjusted.^11^, ^12^ Stereotactic radiotherapy (SRT) uses fewer segments, delivers high single doses, and precisely targets tumors. The radiation dose rapidly decreases at the periphery of the target area, minimizing damage to surrounding normal tissues and cells.^13^ Initially applied to the head, the extension of SRT to the body is known as stereotactic body radiotherapy (SBRT).^14^ In 2015, Zhang et al. demonstrated that SBRT could achieve clinical outcomes comparable to surgery in patients with early stage non‐small cell lung cancer (NSCLC), leading to its specific definition as stereotactic ablative radiotherapy (SABR).^15^ Image‐guided radiation therapy (IGRT) is a four‐dimensional RT technique that uses advanced imaging equipment to monitor tumors and normal organs in real time before and during treatment.^16^ This allows the adjustment of treatment conditions based on changes in organ position, thereby reducing systematic and positional errors. Adaptive radiotherapy (ART), an offshoot of IGRT, uses computer technology to dynamically modify the RT plan in real time based on anatomical changes in the tumor and surrounding tissues during treatment, significantly improving the accuracy of RT.^17^, ^18^ Proton radiotherapy (PRT) uses proton beams as the radiation. The main difference between proton beams and high‐energy X‐rays is the dose distribution in the body. Proton beams release a minimal dose throughout their path; however, at the end of their range, they release all their energy, forming the so‐called Bragg peak. This property allows for more precise control of the radiation dose, thereby reducing damage to the surrounding healthy tissues.^19^, ^20^, ^21^ Heavy ion radiotherapy (HIRT), which uses heavy particles such as carbon ions, exhibits a similar dose distribution to protons but with higher relative biological effectiveness (RBE). It can damage the DNA of tumor cells more effectively, especially in hypoxic or radioresistant tumors.^22^, ^23^

### 1.2 Fundamentals of RT

Understanding the therapeutic mechanisms of RT begins with its underlying physical and biological principles.

#### 1.2.1 Role of ionizing radiation

The basis of both the direct and indirect biological effects of ionizing radiation involves the ionization and excitation of biological macromolecules. When ionizing radiation acts directly on biological macromolecules (e.g., nucleic acids, proteins, and enzymes), it may lead to structural changes and loss of biological activity. This damage to biological macromolecules is considered the direct effect of ionizing radiation. Additionally, ionizing radiation interacts with water molecules to generate free radicals that can attack biological macromolecules, thus inducing physical and chemical changes and promoting apoptosis or cellular aging. This is the indirect effect of ionizing radiation.

#### 1.2.2 Cellular damage from ionizing radiation

##### 1.2.2.1 DNA damage and repair

Radiation can induce both lethal and sublethal effects. Sublethal effects resulting from improper repair can lead to genomic instability and increase the risk of radiation‐induced malignancies. In contrast, DNA is the primary target of lethal effects. DNA damage can occur through base modifications, single‐ and double‐strand breaks, and DNA crosslinking, with double‐strand breaks (DSBs) being the most lethal. Single‐strand breaks (SSBs) are typically repaired rapidly, whereas the DSB repair is more dependent on the cellular repair capacity.^24^, ^25^, ^26^

##### 1.2.2.2 Damage to other macromolecules

Radiation can oxidize and dehydrogenate proteins, leading to protein inactivation, structural changes, chemical chain breaks, and protein crosslinking or polymerization, which disrupt normal protein function.^27^, ^28^ Additionally, radiation can break and inactivate glycan chains, which are critical components of the membrane signal transduction system.^29^ Radiation can also induce lipid peroxidation in membranes, leading to increased lipoxygenase and cyclooxygenase activity. This in turn promotes the production of inflammatory mediators, including prostaglandins, thromboxanes, and leukotrienes, which act on endothelial cells and leukocytes to trigger inflammatory responses. Furthermore, radiation induces gene expression that amplifies inflammatory responses.^30^ Additionally, radiation can damage membrane structures and alter membrane‐bound enzymes, receptors, and ion channels, thereby impairing the ability of the cell membrane to maintain its normal function. Mitochondrial membrane damage also disrupts energy metabolism.^31^

#### 1.2.3 Immunomodulatory effects of ionizing radiation

Ionizing radiation also exerts immunomodulatory effects by inducing various immunogenic and phenotypic changes in malignant cells, remodeling the tumor microenvironment (TME), and promoting or suppressing both innate and adaptive immune responses.^32^ Additionally, immune response has been linked to the abscopal effect (non‐radiation‐induced tumor regression following RT).^33^

#### 1.2.4 Bystander effect of ionizing radiation

The bystander effect refers to the phenomenon in which cells adjacent to irradiated cells also exhibit radiation‐induced stress responses. These responses include induction of gene expression, gene mutations, micronucleus formation, cell differentiation, cell apoptosis, and malignant transformation.^34^

#### 1.2.5 Biological basis of fractionated RT: the “4Rs”

Early clinical practice demonstrated that dividing the total radiation dose into more fractions could reduce adverse effects. The response of cells to fractionated RT is primarily characterized by four key aspects, known as the “4Rs.” These include the repair of sublethal damage, redistribution within the cell cycle, reoxygenation of tumors, and repopulation of cells in tissues.^35^ These four mechanisms form the biological foundation of fractionated RT.

## 2 THE ROLE OF RT IN THE TREATMENT OF TUMORS

RT plays a crucial role in tumor treatment. This includes radical, adjuvant, and palliative RT (Table 1), all of which are essential components of the overall treatment strategy and should not be underestimated.

**TABLE 1:** Clinical evidence and application of different treatment modalities in RT.

Type	Clinical Indication	Primary Efficacy	Ref.
Radical RT	Nasopharyngeal carcinoma, SCLC, and HL	For tumors with high radiosensitivity, RT can achieve curative effects on its own	^37^, ^38^, ^39^
	Early prostate cancer and cervical cancer	The cure rate with radical RT alone is comparable to that of surgery	^40^, ^42^
	Tumors are located in areas that are difficult to resect (such as prostate cancer or anorectal cancer)	Radical RT can be used to precisely target the tumor, thereby preserving vital organ function while minimizing excessive damage to normal tissues	^40^, ^41^
Neoadjuvant RT	Locally advanced rectal cancer	The German CAO/ARO/AIO‐94 trial showed that neoadjuvant CRT significantly reduced the rate of local recurrence (6% vs. 13%) and minimized treatment‐related toxicity	^46^
	Locally advanced esophageal cancer	Neoadjuvant CRT has been shown to improve the surgical resection rate and survival	^47^
	Locally advanced pancreatic cancer	Neoadjuvant CRT can convert some unresectable pancreatic cancers into resectable ones	^48^
Adjuvant RT	NSCLC (High‐risk factors confirmed by postoperative pathology)	Adjuvant RT can reduce the risk of local recurrence	^50^
	Radical prostatectomy	The SWOG 8794 trial showed that adjuvant RT can significantly improve local control rates and biochemical DFS	^52^
Palliative RT	Bone metastasis pain	A single dose of RT at 8 Gy can provide pain relief for more than 70% of patients	^57^
	Brain metastases	Whole‐brain RT is the standard palliative treatment for multiple brain metastases, improving neurological symptoms and quality of life	^58^
	Malignant spinal cord compression	Prompt palliative RT can prevent permanent nerve damage, reduce pain, and improve motor function	^60^
	Bleeding caused by malignant tumors	RT can effectively control bleeding caused by malignant tumors in the lungs, bladder, and digestive tract	^61^, ^64^

### 2.1 Radical RT

Radical RT is aimed at curing tumors. It delivers a sufficiently high dose to precisely target the tumor with the intention of eliminating tumor cells and thus achieving long‐term disease‐free survival (DFS). The primary goal is to control or eradicate the tumor rather than to simply relieve symptoms or improve the quality of life.^36^

RT alone can achieve curative effects in tumors with high radiosensitivity, such as nasopharyngeal carcinoma, small cell lung cancer (SCLC), and HL.^37^, ^38^, ^39^ In cases where tumors are located in areas that are difficult to resect, such as in prostate or anorectal cancer, radical RT can be used to precisely target the tumor, thereby preserving vital organ function while minimizing excessive damage to normal tissues.^40^, ^41^ Radical RT is usually administered to patients with early or locally advanced tumors. In the early stages of cancers, such as prostate and cervical cancers, the cure rate with radical RT alone is comparable to that with surgery.^40^, ^42^

### 2.2 Adjuvant RT

Adjuvant RT includes neoadjuvant and adjuvant RT. Neoadjuvant RT refers to the administration of RT before surgery to reduce tumor size and stage, improve surgical resection probability,^43^ and reduce the risk of postoperative recurrence.^44^ For example, neoadjuvant chemoradiotherapy (CRT) is the standard treatment in locally advanced rectal cancer.^45^ The German CAO/ARO/AIO‐94 trial showed that compared with postoperative CRT, neoadjuvant CRT significantly reduced the rate of local recurrence (6% vs. 13%) and minimized treatment‐related toxicity.^46^ Neoadjuvant CRT improves surgical resection rates and survival in locally advanced esophageal cancer.^47^ Similarly, it may improve surgical resection rates and survival in locally advanced pancreatic cancer. Studies have shown that neoadjuvant CRT can convert unresectable pancreatic cancers into resectable ones in some cases.^48^

Adjuvant RT refers to the administration of RT after surgery to eliminate residual microscopic lesions, reduce the risk of local recurrence, and improve long‐term survival. Adjuvant RT has become the standard treatment following breast‐conserving surgery, significantly reducing the rate of local recurrence and improving overall survival (OS).^49^ In patients with NSCLC, especially those with high‐risk factors as confirmed by postoperative pathology, adjuvant RT can reduce the risk of local recurrence.^50^ After radical prostatectomy, adjuvant RT can reduce the risk of biochemical recurrence and prolong DFS.^51^ The SWOG 8794 trial showed that adjuvant RT significantly improves local control rates and biochemical DFS.^52^

### 2.3 Palliative RT

Palliative RT is used to treat incurable tumors. Unlike radical RT, the primary goal of palliative RT is to relieve symptoms and improve quality of life rather than curing the disease.^53^

Common effects of palliative RT include pain relief, control of bleeding, reduction of tumor compression, and improvement of dyspnea.^54^, ^55^ Bone metastasis is a major cause of pain in patients with advanced malignancies. Palliative RT can effectively relieve this pain, improve patient mobility, and reduce the need for analgesics.^56^ Studies have shown that a single dose of RT at 8 Gy can provide pain relief in more than 70% of patients.^57^ In patients with brain metastases or spinal cord compression, palliative RT can reduce symptoms, such as headache, seizures, and movement disorders. Whole‐brain RT is the standard palliative treatment for multiple brain metastases, improving neurological symptoms and quality of life.^58^ In patients with a small number of brain metastases, SRT can deliver precise high‐dose irradiation while minimizing exposure to the normal brain tissue.^59^ Prompt palliative RT can prevent permanent nerve damage, reduce pain, and improve motor function in patients with malignant spinal cord compression, which is an acute condition.^60^ Additionally, palliative RT can effectively control bleeding caused by malignant tumors in the lungs, bladder, and digestive tract.^61^, ^62^, ^63^, ^64^ RT can relieve symptoms and improve the ability to breathe and eat in patients with airway obstruction or dysphagia resulting from malignant tumor compression.^65^, ^66^

## 3 COMBINATION OF RT AND OTHER TREATMENTS

RT plays a crucial role as a localized and precise therapeutic modality in the comprehensive treatment of tumors when combined with other strategies, such as chemotherapy, targeted therapy, and immunotherapy (Table 2). The combined approaches maximize the benefits of each treatment and overcome the limitations of individual treatments, thereby improving therapeutic outcomes significantly.

**TABLE 2:** Representative clinical trials of RT combined with chemotherapy, targeted therapy, and immunotherapy.

Types of Combined Therapy	Test Name	Cancer Types	Key Findings	Ref.
CRT	PORTEC‐3 trial	High‐risk endometrial cancer	Compared with RT alone, CRT demonstrated significant benefits in both 5‐year OS rate (81.4% vs 76.1%) and PFS rate (76.5% vs 69.1%)	^73^
	INTERLACE trial	Locally advanced cervical cancer	The 5‐year PFS rate in the group who received chemotherapy followed by cCRT (72%) was superior to the group treated with CRT (64%)	^74^
	CAO/ARO/AIO‐12 and OPRA trials	Locally advanced rectal cancer	A higher ORR with CRT followed by chemotherapy compared to chemotherapy followed by CRT	^75^
Combined RT and Targeted Therapy	LAURA trial	Unresectable stage III EGFR‐mutated NSCLC	Osimertinib after CRT significantly prolonged median PFS (39.1 months vs 5.6 months)	^87^
	RECELL trial	Unresectable stage III EGFR‐mutated NSCLC	Erlotinib combined with RT significantly improved PFS compared to cCRT (24.5months vs. 9.0 months)	^88^
	NROGC‐002 trial	EGFR‐mutated oligo‐organ metastatic NSCLC	EGFR‐TKI plus concurrent TRT had significantly better median PFS (17.1 months vs. 10.6 months) compared to TKIs alone	^89^
iRT	Jinming Yu et al.trial	ES‐SCLC	Combining adebrelimab with chemotherapy and sequential thoracic RT significantly improved the prognosis of patients	^131^
	Helen J. Ross et al.trial	Unresectable stage III NSCLC	Safety and efficacy of atezolizumab induction and consolidation therapy before and after cCRT	^133^
	SPRINT trial	Unresectable LA‐NSCLC and PD‐L1 TPS ≥50%	Pembrolizumab followed by RT (without chemotherapy) was well‐tolerated and effective for patients	^134^
	Markussen A et al.trial	Metastatic biliary tract cancer	SBRT/nivolumab/ipilimumab group had a higher rate of clinical benefit (complete response + partial response + stable disease) than the SBRT + nivolumab group (31.0% vs. 10.5%)	^135^

### 3.1 Chemoradiotherapy

#### 3.1.1 Mechanisms of chemoradiotherapy

Chemotherapy is a systemic treatment in which drugs circulate through the blood to reach the entire body, thus interfering with tumor cell division and replication and ultimately inducing tumor cell death. The effectiveness of chemotherapy depends on several factors including tumor pathology, drug mechanism, dosage, method of administration, and individual patient differences. The principle behind chemoradiotherapy (CRT) is based on different mechanisms that complement each other to increase the overall antitumor effect.^67^ RT is a localized treatment that uses radiation to kill tumor cells, whereas chemotherapy is a systemic treatment that uses chemical agents to target and eliminate tumor cells throughout the body.

The mechanisms underlying the synergistic effects of CRT involve several factors. First, the sensitivity of tumor cells to RT depends on the cell cycle phase. Some chemotherapeutic agents, such as temozolomide, can induce the G2/M phase in tumor cells, making them more sensitive to RT and increasing its effectiveness.^68^ Additionally, RT‐induced DNA damage can be repaired through tumor cell repair mechanisms. Certain chemotherapeutic agents, such as platinum‐based drugs, can inhibit DNA repair mechanisms, including homologous recombination (HR) and nonhomologous end‐joining (NHEJ), leading to the RT‐induced accumulation of DSBs and promotion of tumor cell death.^69^ Furthermore, RT and chemotherapy induce apoptosis via different mechanisms. RT primarily causes apoptosis by directly damaging the DNA of tumor cells, whereas chemotherapeutic agents synergistically activate apoptotic signaling pathways and inhibit the expression of anti‐apoptotic proteins, thereby promoting apoptosis.^70^

#### 3.1.2 Clinical applications of CRT

For locally advanced tumors such as nasopharyngeal, rectal, esophageal, lung, and cervical cancers, concurrent chemoradiotherapy (cCRT) can significantly improve local control and therapeutic efficacy. For example, before the PACIFIC study, cCRT was the first‐line therapy for locally advanced NSCLC (LA‐NSCLC). In a study of 64 patients with advanced esophageal cancer, the objective response rate (ORR) and 2‐year survival rate were higher in the group receiving CRT than in the group receiving RT alone (ORR: 84.38% *vs*. 71.88%; 2‐year survival rate: 65.63% *vs*. 40.63%, *P* < 0.05).^71^ Irinotecan plus S‐1 combination chemotherapy (IRIS) combined with concurrent RT for the treatment of recurrent rectal cancer showed a complete response, partial response, and stable disease rates of 8.3%, 36.1%, and 41.7%, respectively, in 36 patients, and symptom response rate of 81.0% in 21 patients with clinical symptoms.^72^ In the PORTEC‐3 trial, the 5‐year OS rate for patients with high‐risk endometrial cancer who were treated with CRT was 81.4% compared to 76.1% in those treated with RT alone. The 5‐year progression‐free survival (PFS) rates were 76.5% and 69.1%, respectively, indicating a significant survival benefit.^73^

However, the order, dose, and combination modes of CRT remain to be explored. In the INTERLACE trial, the 5‐year PFS rate in patients with locally advanced cervical cancer who received chemotherapy followed by cCRT was 72% compared to 64% in the patients treated with CRT. The 5‐year OS rates were 80% and 72%, respectively.^74^ In patients with locally advanced rectal cancer, a pooled analysis of the CAO/ARO/AIO‐12 and OPRA trials reported a higher ORR with CRT followed by chemotherapy compared to that with chemotherapy followed by CRT.^75^ Regarding the optimal RT regimen of cCRT in locally advanced esophageal squamous carcinoma, a study led by our institution compared the total dose and irradiation field of RT: 59.4 Gy in the high‐dose (HD) group versus 50.4 Gy in the standard‐dose (SD) group and involved field irradiation (IFI) group versus elective nodal irradiation (ENI) group. Compared with the SD group, the HD group showed improved PFS (25.2 vs. 18.0 months) but not significantly improved OS. The HD+IFI group had the best survival rate, whereas the SD+IFI group had the worst prognosis.^76^

There were significant individual differences in responses to cCRT. Therefore, clinical decisions must consider the patient's condition and physical status. Furthermore, although combination therapy can enhance treatment efficacy, it may also increase the complexity and severity of side effects.^77^, ^78^, ^79^, ^80^ Therefore, close monitoring during the treatment process is essential, along with timely evaluation and adjustment of the treatment plan to optimize efficacy while minimizing adverse effects.

### 3.2 Combined RT and targeted therapy

#### 3.2.1 Mechanisms of combined RT and targeted therapy

Targeted therapies, including small‐molecule tyrosine kinase inhibitors (TKIs), monoclonal antibodies, and endocrine therapeutic drugs, inhibit tumor growth by specifically blocking key molecules, genes, or proteins involved in cancer signaling pathways.^81^ Targeted drugs include epidermal growth factor receptor (EGFR) inhibitors, vascular endothelial growth factor receptor (VEGFR) inhibitors, and human epidermal growth factor receptor 2 (HER2) inhibitors.

The principle behind combining RT and targeted therapy is that RT kills tumor cells primarily through radiation, whereas targeted therapy inhibits tumor growth by targeting specific molecular markers on the surface of the tumor cells. They act on tumor cells through different mechanisms and exert synergistic antitumor effects. The mechanism underlying the synergistic effect of RT and targeted therapy involves several aspects. First, RT induces DNA DSBs, which trigger the accumulation of targeted therapeutic agents and thus impedes DNA damage repair and suppresses tumor resistance to RT. For example, poly ADP‐ribose polymerase (PARP) inhibitors can block DNA repair, thereby increasing the sensitivity of tumor cells to RT.^82^ Studies have shown that the combination of PARP inhibitors and RT can significantly improve the survival rates of patients with locally advanced prostate cancer.^83^ In addition, RT can prevent tumor cells from entering phases of the cell cycle that are less sensitive to radiation, while targeted therapy further enhances this effect by inhibiting key cell cycle proteins, thereby improving the effectiveness of RT.^84^ Furthermore, RT also induces the expression of angiogenic factors, thereby promoting angiogenesis. However, targeted therapies, such as VEGFR inhibitors, can inhibit angiogenesis and reduce tumor oxygenation, enhancing the effects of RT.^85^ Additionally, both RT and targeted therapy affect the epigenetic regulation of tumor cells. RT can induce DNA methylation, whereas targeted therapy can inhibit epigenetic enzymes, alter chromatin structure, and enhance RT sensitivity.^85^

#### 3.2.2 Clinical application of combined RT and targeted therapy

In the treatment of many tumors, a combination of RT and targeted therapy can promote local tumor control, reduce mortality, and does not increase the toxicity associated with RT.^86^ For example, RT combined with targeted therapy can relieve the symptoms and prolong the survival of patients with lung cancer. The LAURA study was the first phase III clinical trial to explore targeted therapies for unresectable stage III *EGFR*‐mutated NSCLC. The results demonstrated that the median PFS of osimertinib after CRT was 39.1 months, compared to 5.6 months in the CRT group, representing a significant improvement in PFS.^87^ The RECELL trial led by our institution included 40 patients with unresectable stage III *EGFR*‐mutated NSCLC. Erlotinib combined with RT significantly improved PFS compared to cCRT (24.5 vs. 9.0 months, *P* < 0.001), with ORRs of 70% and 61.9%, respectively. This study achieved the primary endpoint of PFS, indicating that EGFR‐TKIs combined with RT is superior to cCRT in treating unresectable stage III *EGFR*‐mutated NSCLC.^88^ Another multicenter clinical trial led by our institution (NROGC‐002) showed that EGFR‐TKI plus concurrent thoracic radiotherapy (TRT) in patients with *EGFR*‐mutated oligo‐organ metastatic NSCLC had significantly better median PFS (17.1 vs. 10.6 months) and median OS (34.4 *vs*. 26.2 months) than those treated with TKIs alone.^89^ A real‐world study demonstrated that patients with unresectable stage III *ALK*‐positive NSCLC showed a significant improvement in PFS when treated with consolidated ALK‐TKIs compared to durvalumab or observation after CRT.^90^ In addition, the combination of EGFR inhibitors with RT has become a well‐established clinical practice in head and neck cancer. In patients with locoregionally advanced squamous cell carcinoma of the head and neck, the addition of cetuximab to RT significantly improves locoregional control and OS. Beyond acneiform rash and infusion reactions, it does not increase the incidence of common toxic effects associated with head and neck RT. Therefore, it is often used as an alternative for patients unsuitable for receiving cisplatin chemotherapy.^91^, ^92^ The anti‐VEGF agent bevacizumab has anti‐angiogenic properties in lung cancer. Theoretically, it may exert radiosensitizing effects by inhibiting the formation of abnormal tumor blood vessels and enhancing oxygenation.^93^ Two studies from the Sun Yat‐sen University Cancer Center indicated that combining bevacizumab with fractionated SRT (FSRT) significantly improved intracranial control and quality of life in patients with NSCLC and brain metastases, with manageable toxicity. This approach has substantial potential for patients who are unsuitable for surgery or whole‐brain RT. Future validation of its long‐term efficacy will require larger‐scale clinical trials.^94^, ^95^

However, the combination of RT and targeted therapy also carries risks and the response to this combination therapy varies greatly among patients. Therefore, it is necessary to create individualized treatment plans that consider the patient's disease, physical status, and molecular and biological characteristics. The optimal combination of RT and targeted therapy is still being explored, and further clinical studies are required to identify the most effective treatment combinations and dose strategies. These studies will help improve efficacy while minimizing toxicity.

### 3.3 Combined RT and immunotherapy

In recent years, numerous studies have shown that RT exerts antitumor effects by regulating both local and systemic immune responses.^96^, ^97^, ^98^, ^99^ With the development and widespread use of immune checkpoint inhibitors (ICIs), the immunomodulatory effects of RT and the synergistic potential of combined RT and immunotherapy (iRT) have gained increasing attention in clinical practice.

#### 3.3.1 Mechanisms of iRT

In addition to its immunosuppressive effects,^100^, ^101^ RT can also activate the immune system, providing a theoretical basis for combining immunotherapy with various forms of RT. Studies have shown that RT can remodel the TME to enhance sensitivity to immunotherapy.^102^ Radiation‐induced DSBs increase the expression of programmed death ligand‐1 (PD‐L1) in tumor cells through the ATM/ATR/Chk1 pathway.^103^ In addition to directly damaging tumor cells, RT triggers a series of immune‐mediated antitumor effects.^104^, ^105^, ^106^, ^107^, ^108^ For example, RT can induce immunogenic cell death and antigen release^109^ and upregulate MHC‐I molecules on tumor cells.^110^ Additionally, RT stimulates potent antitumor responses by influencing the cancer–immunity cycle.^104^, ^111^ These effects include enhanced release and presentation of tumor antigens,^112^, ^113^ promotion of immune cell initiation and activation,^114^, ^115^ maturation of dendritic cells, increase in the density of tumor‐infiltrating lymphocytes,^116^, ^117^ enhancement of T‐cell recognition by tumor cells, and promotion of specific T‐cell infiltration and migration.^114^, ^118^ Combining RT with anti‐PD‐L1 antibodies can synergistically reduce the accumulation of tumor‐infiltrating myeloid‐derived suppressor cells (MDSCs) and alter the tumor immune microenvironment.^119^ Furthermore, the combination of dual ICIs and RT can activate nonredundant immune mechanisms. In this combination, RT enhances the diversity of the T‐cell receptor repertoire, anti‐cytotoxic T lymphocyte‐associated antigen‐4 (CTLA‐4) primarily suppresses regulatory T cells (Tregs), and anti‐PD‐L1 reverses T‐cell exhaustion.^120^ This reprogrammed TME can transform “cold” tumors, which have low immune cell infiltration, into “hot” tumors, which have abundant lymphocyte infiltration. This process has been widely studied and recognized.^121^, ^122^

#### 3.3.2 Clinical applications of iRT

iRT can effectively enhance therapeutic effects by expanding the strengths and complementing the disadvantages of RT and immunotherapy. In 2021, Theelen et al. clinically confirmed for the first time that iRT can enhance the abscopal effect, potentially doubling the clinical efficacy in patients with advanced NSCLC.^123^ Furthermore, the administration of pembrolizumab after RT significantly prolonged the median PFS (4.4 *vs*. 2.1 months, *P* = 0.019) and median OS (10.7 *vs*. 5.3 months, *P* = 0.026) compared to patients who did not receive RT.^124^ Additionally, iRT has shown great success in treating melanoma and other solid tumors.^125^, ^126^, ^127^, ^128^

Currently, three main forms of iRT are applied clinically: sequential treatment with RT followed by immunotherapy, induction treatment with immunotherapy followed by RT, and simultaneous treatment with RT and immunotherapy.^129^ Therefore, one critical issue requiring attention is the optimal timing of the simultaneous or sequential combination.^130^ Recently, a team led by Yu demonstrated that combining adebrelimab with chemotherapy and sequential thoracic RT significantly improved the prognosis of patients with extensive‐stage small‐cell lung cancer (ES‐SCLC).^131^ The median PFS was 10.1 months, and the median OS was 21.4 months, with 1‐year and 2‐year OS rates of 74.1% and 39.7%, respectively. Furthermore, this treatment was well tolerated, with the incidence of grade ≥3 pneumonitis being only 6%.^131^ A prospective, single‐arm, multicenter phase II clinical trial demonstrated the efficacy and safety of durvalumab after cCRT for unresectable stage III NSCLC, achieving a 1‐year PFS rate of 75%.^132^ Ross et al. demonstrated the safety and efficacy of atezolizumab induction and consolidation therapy before and after cCRT in patients with unresectable stage III NSCLC.^133^ The SPRINT study found that pembrolizumab followed by RT (without chemotherapy) was well tolerated and effective in patients with unresectable LA‐NSCLC and a PD‐L1 tumor proportion score ≥50%.^134^ A phase II clinical study on metastatic biliary tract cancer evaluated the clinical efficacy and safety of dual immunotherapy (nivolumab combined with ipilimumab) plus SBRT and showed that the SBRT/nivolumab/ipilimumab group had a higher rate of clinical benefit (complete response + partial response + stable disease) than the SBRT + nivolumab group (31.0% *vs*. 10.5%). However, there was no significant improvement in the median OS (5.4 *vs*. 4.7 months).^135^ Additionally, the optimal timing of iRT is not the only noteworthy factor to consider, and the doses of RT and immunotherapy are equally important. A phase I clinical trial evaluated the feasibility of fractionated dose escalation of hypofractionated radiotherapy (HFRT) with concurrent chemotherapy and subsequent consolidation immunotherapy. This study used split‐course HFRT and Hypoboost, and the results showed that the regimen, from a single dose of 5 Gy to a total dose of 60 Gy, was well tolerated and showed promising results in terms of ORR and survival.^136^ Notably, many clinical trials on iRT are currently underway. These studies will provide valuable data to help select the optimal iRT modality.

Although iRT is a promising therapeutic approach against tumors, not all clinical trials have demonstrated that this combination improves survival. A phase III trial involving prostate cancer showed no significant difference in OS between the RT combined with ipilimumab group and the RT‐only group of patients who progressed after docetaxel treatment.^137^ Preclinical evidence suggests that the combination of HFRT and ICIs significantly enhances tumor control.^138^ However, a clinical trial on head and neck squamous cell carcinoma found that nivolumab plus SBRT failed to improve patient prognosis compared to nivolumab alone.^139^ Additionally, the median PFS of patients with metastatic castrate‐resistant prostate cancer treated with 300 cGy × 10 doses of RT combined with sipuleucel‐T immunotherapy was similar to that of patients who received sipuleucel‐T immunotherapy alone.^140^

Thus, synergy between RT and immunotherapy depends on several factors. RT has a dual immunomodulatory effect.^101^, ^141^ Although RT can stimulate the immune system, it can also suppress immune responses at certain doses using specific fractionation schemes.^142^, ^143^ This necessitates careful consideration of how to optimize iRT, which presents many challenges including patient selection, treatment combinations, RT dose and fractionation, management of adverse effects, and timing of immunotherapy interventions. To address these challenges, it is crucial to establish standardized professional guidelines for clinical diagnosis and treatment, ultimately maximizing patient benefits. Based on the current trends, both RT and immunotherapy are moving toward greater individualization, whether used alone or in combination.

In conclusion, RT is a crucial modality in tumor treatment that precisely targets tumors using high‐energy radiation. When combined with other treatments, the cure rate of RT can significantly improve. From a biological perspective, RT can be combined with chemotherapy, targeted therapy, and immunotherapy through mechanisms, such as DNA damage, TME modulation, and immune system activation. As a core component of comprehensive treatment, RT continues to play a key role in the multidisciplinary field of cancer treatments.^144^

## 4 THE BREAKTHROUGHS AND FUTURE OF RT

With the rapid advancement in technology and treatments, RT has entered a new era. The following sections will discuss PRT and HIRT, FLASH radiotherapy (FLASH‐RT), ART, artificial intelligence (AI) and big data, biomarker‐guided RT, combination of RT and emerging treatments, exploration of molecularly targeted RT, and prediction and management of RT related adverse events by focusing on how these technological advancements are driving the development of RT.

### 4.1 PRT and HIRT

RT is a crucial modality in tumor treatment, and PRT and HIRT (particularly carbon‐ion radiotherapy [CIRT]) have garnered significant attention as innovative technologies owing to their excellent dose control and RBE.^145^ PRT, with its unique physical properties, can deliver the maximum dose at the tumor depth while rapidly decreasing beyond the tumor, thereby minimizing damage to normal tissues.^146^ CIRT offers therapeutic advantages for tumors that are challenging to treat using conventional RT owing to its higher RBE and more precise dose distribution.^147^

Recent advances in PRT have focused on improving efficiency and reducing costs. Although the number of PRT centers is increasing worldwide, it remains insufficient compared with the number of conventional X‐ray RT facilities.^148^ Technological innovations such as fixed‐beam RT, which does not rely on large, heavy, and expensive rotating gantries, help meet patient needs.^149^ CIRT has also facilitated significant advances in terms of dose distribution, treatment planning, and beam delivery.^150^

Future development of PRT and HIRT will focus on technological innovation, clinical trials, and international cooperation. In terms of technological innovation, researchers are exploring smaller, lower‐cost, and more effective devices as well as integrated multi‐ion devices to meet treatment needs and gradually replace some photon RT applications.^151^ In clinical trials, the precision of PRT allows for reduced exposure of normal tissues and organs to radiation, thereby minimizing the side effects. For example, PRT reduces genitourinary and gastrointestinal toxicities during prostate cancer treatment. HIRT has demonstrated significant advantages in the treatment of refractory tumors (e.g., recurrent nasopharyngeal carcinoma, chordoma, and glioma).^144^ Hu et al. treated 206 patients with locally recurrent nasopharyngeal carcinoma using CIRT and showed that CIRT substantially improved survival and reduced toxicity.^145^ Although existing clinical studies have shown that PRT and HIRT offer significant advantages over conventional photon RT for certain tumors, more high‐quality randomized controlled trials (RCTs) are needed to clarify the clinical value and potential risks of these therapies against various tumors.^152^ International collaboration can accelerate the development and global application of these technologies by sharing data and experiences.^153^

### 4.2 FLASH‐RT

FLASH‐RT has revolutionized RT with its unique ultrahigh dose rate (UHDR), typically ≥40 Gy/s. Compared with conventional RT, FLASH‐RT can maintain its tumor cell‐killing effects while significantly reducing damage to normal tissues, a phenomenon known as the FLASH effect.^154^

The current understanding of the effect of FLASH‐RT cannot be explained by a single mechanism. Mechanisms, such as oxygen depletion,^155^ free radical reactions, DNA damage and repair, and TME regulation, may collectively protect normal tissues.^156^ At UHDRs, FLASH‐RT can rapidly deplete local oxygen and induce a transient hypoxic state, which offers a protective effect on normal tissues while having less impact on tumors.^157^ Additionally, the effects of FLASH‐RT on DNA damage and repair have gained popularity. Some studies indicate that FLASH‐RT may reduce SSBs, whereas its effect on DSBs is less.^158^ Regarding its effect on immune response, FLASH‐RT may influence antitumor immunity by altering the immune cell composition in the TME, particularly by recruiting T lymphocytes.^159^ Moreover, it has also been suggested that FLASH‐RT may protect circulating immune cells, thus reducing the overall destruction of the immune system.^146^ Additionally, an innovative proton therapy technique named spot‐scanning proton arc therapy + FLASH (SPLASH) has been developed. SPLASH combines the multi‐angle, dynamic irradiation of spot‐scanning proton arc therapy with the UHDR characteristics of FLASH‐RT. This integrated technology enables high‐dose irradiation in extremely short timeframes (milliseconds) while ensuring precise tumor targeting, thus driving RT toward greater efficiency and minimal invasiveness.^160^

The clinical translation of FLASH‐RT is progressing steadily from early case trials to the preliminary clinical trial phase. In 2019, the first patient with radioresistant cutaneous lymphoma successfully underwent FLASH‐RT in accelerated electron mode,^161^ providing evidence of the technical feasibility of subsequent standardized studies. In 2022, the first UHDR proton FLASH‐RT clinical trial, FAST‐01, was completed. This trial evaluated the feasibility and safety of treating symptomatic extremity bone metastases^162^, ^163^ and demonstrated the feasibility of the clinical workflow and a favorable preliminary safety profile for FLASH‐RT. A follow‐up study, FAST‐02, is currently underway to assess the efficacy and toxicity of proton FLASH‐RT in the treatment of sternal bone metastases.^164^ Currently, international multidisciplinary experts have reached a consensus andformulated a scientifically sound and reasonable plan for the clinical trial design of the next phase of proton FLASH‐RT.^165^

In recent years, a Chinese team has made significant progress in FLASH‐RT research through preclinical animal trials, and the effects of FLASH‐RT have been validated in mouse models under UHDR X‐ray conditions, thus demonstrating its protective effects on normal tissues, including the lungs, brain,^166^ and intestines.^167^ A compact device that supports this technology was developed.^168^ Simultaneously, China has advanced both the clinical application and development of technological platforms for FLASH‐RT. Established in 2019, the Platform for Advanced Radiotherapy Research (PARTER) is the first facility to conduct FLASH experiments using megavoltage X‐rays. With its high‐precision dose control, the platform supports preclinical studies, including animal models, thereby ensuring data reliability and reproducibility.^169^ With regard to clinical translation, a multicenter clinical trial of UHDR electron beam radiotherapy (e‐Flash), initiated by the West China Hospital, Sichuan University, reported on October 14, 2025 that the treatment for five patients with skin cancer had been completed. Pending confirmation of safety and efficacy, plans are underway to enroll 63 additional patients in a confirmatory trial.^170^ This study provides foundational data for building clinical evidence on FLASH in China. Regarding the methodology and technology, a team at Peking University proposed an optimization scheme for the dose and dose rates in proton FLASH‐RT. This approach maximizes the UHDR coverage of critical organs while maintaining the dose measurement metrics in proton pencil beam‐scanning FLASH‐RT,^171^ thus providing a theoretical foundation for optimizing clinical planning in proton FLASH‐RT. Concurrently, several teams including the Shanghai Advanced Research Institute, Chinese Academy of Sciences have proposed an integrated design for FLASH treatment facilities based on proton linear accelerators.^172^ This design aims to achieve a stable UHDR proton beam output, offering a potential hardware solution for clinical FLASH implementations.

Despite the positive results of preclinical studies, the clinical application of FLASH‐RT faces many challenges. Different tumor sites and types have varying requirements for dose distribution and penetration, which raises the question of optimizing FLASH‐RT to meet clinical needs. This requires comprehensive optimization of equipment design, radiation source selection, and treatment protocols. Additionally, determining the optimal parameters for FLASH‐RT, including total dose, dose rate, irradiation time, and fractionation pattern, remains a key area for future research.^147^

### 4.3 ART

ART has become a key development area in the field of RT as an innovative treatment. The core of ART lies in real‐time monitoring of changes in the tumor and surrounding tissues, allowing for dynamic adjustment of the treatment plan to enhance accuracy and effectiveness.^148^ The emergence of this treatment has marked a shift in RT from static treatment to dynamic and personalized treatment.

ART, when combined with advanced imaging techniques such as IGRT, has enabled the real‐time monitoring of tumor location and shape. With daily imaging feedback, ART can adjust the treatment plan to ensure that the tumor remains within the radiation field.^149^ Magnetic resonance‐guided adaptive radiation therapy (MRgART) provides ART with superior soft‐tissue contrast and more precise tumor localization. MRgART allows for real‐time monitoring of tumors and normal tissues, offering ART more precise image guidance.^149^ The MRI‐guided linear accelerator (MR‐Linac) is an ideal platform for MRgART. This hybrid system integrates an MRI scanner and a therapeutic linear accelerator into a single device. It first acquires MRI images, then completes treatment planning and protocol verification while the patient remains in the treatment position and finally delivers radiation under real‐time MRI guidance.^173^, ^174^ This significantly streamlines and accelerates the RT workflow. The MR‐Linac also substantially reduces the risk of treatment‐related toxicities. In a large‐scale clinical cohort study, the incidence of RT‐related grade 3 toxicity across the entire cohort was only 1.4%.^175^ Additionally, AI has enhanced ART by providing powerful data‐processing capabilities, allowing for faster and more accurate treatment plan adjustments.^150^

Although ART has made remarkable progress, its development presents new opportunities and persistent challenges. Advancements in multimodal image fusion technology will provide more precise information on tumor localization and the biological features for ART.^151^ However, ART will increasingly rely on AI to automate its plans. Moreover, large‐scale RCTs are necessary to validate the efficacy of ART. These trials will provide strong evidence supporting the clinical application of ART.^152^ Despite the significant clinical advantages of ART, its high cost limits its widespread use. Future studies should explore methods to reduce costs and make this advanced treatment more accessible to patients.^153^

### 4.4 AI and big data

With the continuous advancement of information technology, AI and big data have become increasingly sophisticated in the RT field. Currently, the application of AI in RT primarily focuses on three stages: treatment preparation, execution, and outcome evaluation.

Accurate mapping of organs at risk (OARs), tumor targets, and dose design are essential in the treatment preparation phase.^176^ However, this process has become more complex and time‐consuming with increasing RT precision. For OARs, the widely used deformable medical image registration technique can distinguish organs or tissues of similar intensity, offering an improvement over earlier automatic segmentation methods.^154^ The introduction of AI has further enhanced the accuracy and efficiency of automatic OAR outlining. In recent years, several studies have reported the use of deep learning in image segmentation.^157^, ^158^ For example, Xiao et al. constructed an automatic segmentation model based on 2D U‐net and 3D U‐net, which excelled in segmenting OARs such as the bladder, small bowel, and rectum, significantly reducing the outlining time.^158^ For tumor outlining, tumor boundaries often lack clear features and must be determined by integrating multiple imaging modalities, clinical features, and the clinical experience of the radiotherapists. Currently, AI requires further development for automatic tumor delineation. However, AI‐based outlining has shown good specificity with positive external validation results for brain metastases and other tumors with well‐defined boundaries, thus highlighting its potential in delineating tumor areas with clear boundaries.^159^ Additionally, AI can assist physicists in dose design. By learning the contours and dose distribution for CT images, AI can automatically estimate the required dose distribution after inputting a new CT image. The generated treatment plan can be comparable to that produced by RapidPlan.^177^

Cone‐beam CT (CBCT) is currently the most widely used technique for RT during the treatment execution phase. However, the imaging accuracy of CBCT is lower than that of plain CT. Recently, it was found that the accuracy of CBCT can be improved by utilizing AI to learn the differences between CT and CBCT, thus enabling the generation of CT‐like images from CBCT.^178^ For tumors with significant motion, dynamic tracking can effectively improve RT accuracy. Some studies have shown that noninvasive, high‐precision dynamic tracking can be achieved by real‐time tracking with orthogonal X‐ray projection images, with AI compensating for equipment time delays, showing promising results.^179^ AI's ability to process and integrate data is crucial for ART. Furthermore, AI‐assisted treatment preparation significantly shortens the ART workflow time.

In the evaluation stage of RT, AI technologies, particularly deep learning, can effectively integrate dose‐related parameters with non‐dose‐related factors, such as clinical features and genomic data, to identify the key parameters for constructing predictive models. These models can help predict patient outcomes and likelihood of adverse events. This, in turn, enables clinicians to adjust treatment plans, conduct early interventions, improve patient comfort, reduce treatment costs, and ultimately prolong survival. Radiomics is a prime example of leveraging AI to advance precision RT. This approach employs algorithms, such as machine learning, to extract large numbers of quantitative features from medical images at high throughput, subsequently converting them into data suitable for statistical analysis. Radiomics not only closely correlates with genomic data but also has the potential to reveal tumor heterogeneity, making it valuable for patient stratification. Beyond serving as an adjunct to clinical decision‐making, it functions as a research tool to uncover novel molecular disease pathways. This enables further optimization of diagnosis, prediction of disease progression, and improvement in patient outcomes.^180^

In conclusion, AI and big data hold significant potential in RT; however, their application still faces several challenges. First, difficulties persist in integrating big data, collecting comprehensive patient information, and correlating treatment plans with outcomes. Additionally, the reproducibility of automation depends on the image generation process, and improving the consistency of models across different centers and devices remains a critical area for further exploration.

### 4.5 Biomarker‐guided RT

With advances in tumor biology research and omics detection technologies, biomarker‐guided RT has emerged as a key direction for precision RT. It stratifies patients based on genetic, epigenetic, proteomic, and metabolic markers associated with specific physiological states (rather than pathological states) to assess the benefits and risk.^181^ This approach explores individualized pathways for dose adjustment, fractionation optimization, and combinations with systemic therapies. For instance, a Phase II trial used ^18^F‐FMISO (fluoromisonidazole) positron emission tomography (PET) to assess tumor (human papillomavirus‑positive oropharyngeal cancer) hypoxia. Patients without evidence of hypoxia received only 30 Gy of downgraded CRT, which significantly reduced toxicity without compromising survival.^182^ Additionally, researchers have developed a personalized indicator of tumor radiosensitivity, known as the genomic adjusted radiation dose (GARD), by measuring the expression of ten specific genes in the tumor tissue. A higher GARD score indicated better treatment outcomes. This model has been validated for multiple cancer types, including triple‐negative breast cancer and nasopharyngeal carcinoma.^183^

Despite the promising prospects of biomarker‐guided RT, significant challenges remain in clinical practice. First, the clinical utility of many promising biomarkers have not been validated through large‐scale prospective clinical trials. Second, biological characteristics vary widely among individuals. Biomarkers derived from a single biopsy or imaging session may not fully capture all tumor properties. Additionally, the lack of standardized research methodologies leads to poor reproducibility of the findings, hindering their transferability across centers. Finally, the biological mechanisms underlying RT response and toxicity are highly complex. The causal relationships between many biomarkers and RT sensitivity, resistance, or long‐term toxicity remains unelucidated, limiting their precise application. Future research should focus on conducting rigorous prospective clinical trials, establishing standardized analytical protocols, developing more accessible and reliable detection technologies, and advancing fundamental research on the biology of tumor RT.

### 4.6 Combination of RT and emerging treatments

RT possesses unique immune‐activating abilities, including upregulation of MHC class I molecule expression and promotion of tumor antigen release. Therefore, the combination of RT with cellular therapy may enhance the tumor‐killing capabilities.

In a case of rectal cancer with liver metastasis, cytokine‐induced killer cell therapy was administered to the rectal lesion after RT, resulting in remission of the primary lesion and disappearance of liver metastasis. This suggests the potential of combining RT with cytotherapy.^184^ Recent studies have shown that RT combined with chimeric antigen receptor T‐cell (CAR‐T) therapy synergistically enhances antitumor effects in hematologic tumors. RT administered as a bridging therapy after cell collection and before CAR‐T infusion, is safe and effective in improving tumor control in large B‐cell lymphoma.^185^ Furthermore, a small‐sample retrospective study of 14 patients with non‐Hodgkin lymphoma found that RT may be an effective remedial strategy after CAR‐T therapy progression.^186^ In solid tumors, although RT research is still at the preclinical stage, several reports have demonstrated that RT enhances the sensitivity of tumor cells to CAR‐T therapy and promotes CAR‐T infiltration.^187^, ^188^, ^189^ Additionally, CAR‐natural killer cell therapy has shown great promise as an emerging cell therapy when combined with RT in preclinical studies.^190^ In summary, the combination of RT and cell therapy has shown promising results in the treatment of hematological tumors, demonstrating strong synergistic antitumor effects in advanced, refractory, and recurrent cancers. Case reports and preclinical studies have indicated that this combination therapy has great potential in the treatment of solid tumors.

### 4.7 Exploration of molecularly targeted RT

Boron neutron capture therapy (BNCT) is an emerging therapy that combines targeted therapy with RT. It is based on the different distributions of boron compounds (mainly borophenylalanine and sodium mercaptododecaborate) in tumors and normal tissues. When irradiated with neutron beams, these boron compounds undergo a nuclear reaction within tumor cells, generating high linear‐energy transfer rays. The path of these rays is shorter than that of single tumor cells, enhancing their targeting ability.^191^ BNCT is primarily used for recurrent head and neck tumors, malignant gliomas, malignant melanomas, and other tumors that do not respond well or are resistant to conventional treatments. The latest clinical results show that for patients with locally advanced or recurrent head and neck tumors that are unresectable, the ORR of BNCT can reach 80.5%, with a complete response rate of 44.4% and partial response rate of 36.1%.^192^ In patients with recurrent or primary glioblastoma, BNCT significantly prolongs survival.^193^, ^194^ Additionally, BNCT has shown promising therapeutic results against melanoma in several centers.^195^, ^196^ Although significant progress has been made in BNCT, several challenges remain regarding its widespread use. First, the equipment for the neutron beam requires further improvement to enhance the treatment efficiency and stability. Second, selective uptake of boron drugs is critical for the success of BNCT. The absorption efficiency of boron drugs varies among different tumors and tissues, making the development of new boron drugs with higher selectivity essential for broader application of BNCT.

In addition to providing “targets” for RT through molecularly targeted drugs, molecularly targeted probes can also be used to optimize RT regimens and predict treatment outcomes. Previous studies have shown that classical ^18^F‐FDG probe imaging can assess the tumor response to RT and identify residual tumors. Dose adjustment based on FDG PET/CT has been demonstrated to be safe and effective in improving the local control of NSCLC.^197^ Hypoxic tissues are less responsive to RT and probes targeting hypoxia have been shown to predict tumor sensitivity to RT.^197^ However, probes that can directly recognize RT sensitivity are lacking and the issue of nonspecific probe aggregation requires further investigation.

With the ongoing development of new boron drugs and specific molecular probes, molecularly targeted RT is set to further shift tumor therapy toward personalized, highly effective, and minimally invasive methods, ultimately enhancing treatment outcomes for patients with refractory tumors.

### 4.8 Prediction and management of RT‐related adverse events

With advances in high‐precision RT and emerging combined treatment strategies, the clinical value of radiation therapy extends beyond improved tumor control to enhance the capabilities for predicting and intervening in RT‐related adverse events. During the pretreatment phase, comprehensive risk factors should be collected for risk assessment, including the patient's overall health status and comorbidities, prior treatment history, and dosimetric indicators of critical organs within the target area. Furthermore, integrating normal tissue complication probability models enables the calculation of the probability of specific toxicities.^198^ Building on this foundation, AI technologies efficiently integrate and mine multimodal information to generate personalized predictions of adverse reactions such as radiation pneumonitis^199^ and oral mucositis.^200^ This study provides scientific decision support for treatment plan selection, dose settings, and preventive measures.

During the treatment implementation phase, enhanced real‐time monitoring plays a dual role: on one hand, it prevents unintended exposure of critical organs through IGRT and ART, significantly reducing toxicity rates^201^ while on the other hand, integrating patient‐reported outcome side effects and symptom scores into routine workflows markedly improves the sensitivity of early adverse event detection and the efficiency of management.^202^ Following treatment completion, standardized follow‐up is essential for monitoring late toxicities continuously and tracking the quality of life to generate real data. These data can be fed back into the predictive models to facilitate external validation and ongoing refinement.

Overall, AI and big data play crucial roles in enhancing RT efficiency and enabling individualized treatment. The combination of emerging therapeutic tools such as cell therapy and RT has great potential in improving tumor control. Molecularly targeted RT such as BNCT, although still in the developmental stage, offers hope to patients who respond poorly to traditional therapies. These new technologies and therapies provide a promising direction for the future of RT and are important for advancing precision RT.

## 5 CONCLUSION

With advancements in science and technology, tumor treatment has evolved from a singular approach to a multidisciplinary and integrated therapeutic process. As one of the three cornerstone modalities of cancer therapy, radiotherapy (RT) plays an indispensable role in modern treatments. Whether used as radical treatment or in combination with other therapies, RT has demonstrated exceptional efficacy and wide applicability. RT will further enhance precision, efficiency, and personalization through continuous innovation and evolution. New technologies and strategies such as FLASH‐RT, ART, BNCT, iRT, and the combination of RT with cellular therapy will provide new opportunities for a greater number of patients. Additionally, the widespread application of AI and big data will play a crucial role in optimizing the RT process and supporting personalized decision‐making. With continuous progress in medical technology, we believe that tumors will no longer be considered incurable diseases, and RT will continue to play an essential role in this transformative process.

## AUTHOR CONTRIBUTIONS

D.C. designed the review. Y. M., H. Y., and S. S. drafted the manuscript. Y.M. and H.Y. prepared figures. Y. M., H. Y., and S. S. revised the manuscript. All the authors have read and approved the final manuscript.

## CONFLICT OF INTEREST STATEMENT

The authors declare no competing interests.

## ETHICAL STATEMENT

Not applicable.
